# Interferon Gamma Enhances Cytoprotective Pathways via Nrf2 and MnSOD Induction in Friedreich’s Ataxia Cells

**DOI:** 10.3390/ijms241612687

**Published:** 2023-08-11

**Authors:** Riccardo Luffarelli, Luca Panarello, Andrea Quatrana, Francesca Tiano, Silvia Fortuni, Alessandra Rufini, Florence Malisan, Roberto Testi, Ivano Condò

**Affiliations:** 1 Department of Biomedicine and Prevention, University of Rome Tor Vergata, 00133 Rome, Italy; riccardo.luffarelli@icloud.com (R.L.); lucapanarello92@gmail.com (L.P.); a.quatrana@outlook.com (A.Q.); fratiano66@gmail.com (F.T.); silviafortuni@hotmail.com (S.F.); rufini@med.uniroma2.it (A.R.); malisan@med.uniroma2.it (F.M.); roberto.testi@uniroma2.it (R.T.); 2 Departmental Faculty of Medicine and Surgery, Saint Camillus International University of Health and Medical Sciences, 00131 Rome, Italy

**Keywords:** Friedreich’s ataxia, interferon gamma, cytoprotection, Nrf2, MnSOD

## Abstract

Friedreich’s ataxia (FRDA) is a rare monogenic disease characterized by multisystem, slowly progressive degeneration. Because of the genetic defect in a non-coding region of *FXN* gene, FRDA cells exhibit severe deficit of frataxin protein levels. Hence, FRDA pathophysiology is characterized by a plethora of metabolic disruptions related to iron metabolism, mitochondrial homeostasis and oxidative stress. Importantly, an impairment of the antioxidant defences exacerbates the oxidative damage. This appears closely associated with the disablement of key antioxidant proteins, such as the transcription factor nuclear factor erythroid 2-related factor 2 (Nrf2) and the mitochondrial superoxide dismutase (MnSOD). The cytokine interferon gamma (IFN-γ) has been shown to increase frataxin expression in FRDA cells and to improve functional deficits in FRDA mice. Currently, IFN-γ represents a potential therapy under clinical evaluation in FRDA patients. Here, we show that IFN-γ induces a rapid expression of Nrf2 and MnSOD in different cell types, including FRDA patient-derived fibroblasts. Our data indicate that IFN-γ signals two separate pathways to enhance Nrf2 and MnSOD levels in FRDA fibroblasts. MnSOD expression increased through an early transcriptional regulation, whereas the levels of Nrf2 are induced by a post-transcriptional mechanism. We demonstrate that the treatment of FRDA fibroblasts with IFN-γ stimulates a non-canonical Nrf2 activation pathway through p21 and potentiates antioxidant responses under exposure to hydrogen peroxide. Moreover, IFN-γ significantly reduced the sensitivity to hydrogen peroxide-induced cell death in FRDA fibroblasts. Collectively, these results indicate the presence of multiple pathways triggered by IFN-γ with therapeutic relevance to FRDA.

## 1. Introduction

A drastic reduction in expression levels of the frataxin protein underlies the rare monogenic disease Friedreich’s ataxia (FRDA). FRDA patients carry a characteristic mutation of *FXN* gene arising from the homozygous expansion of naturally occurring GAA repeats in a non-coding region [1]. Structural and epigenetic modifications within the affected locus eventually hamper the transcription of this gene [2]. Therefore, the functional activity of frataxin remains, but largely below the level of cellular needs [3]. This protein is a member of the iron–sulphur cluster (ISC) biosynthetic apparatus [4,5], an essential multicomponent complex that provides the cofactor to mitochondrial, cytosolic and nuclear ISC-dependent enzymes [6].

FRDA is characterized by a pathological cascade involving the loss of ISC-dependent activities [7,8], mitochondrial dysfunctions [9,10] and abnormal mitochondrial iron-loading [11]. Strikingly, the impairment of antioxidant defences appears to be consistently associated with FRDA pathophysiology [12,13]. Moreover, FRDA cells are particularly sensitive to various oxidants and stressors able to induce cell death by necrosis and apoptosis [14,15,16]. Notably, the impaired expression and/or activity of nuclear factor erythroid 2-related factor 2 (Nfe2l2/Nrf2) was demonstrated in cellular and animal models of FRDA [13,17,18]. Nrf2 is a transcriptional regulator of genes ensuring cellular antioxidant responses and mitochondrial homeostasis, including mitochondrial biogenesis and mitophagy. It is now widely recognized that because of the proteins encoded by its target genes, Nrf2 is essential for the mechanisms of cytoprotection [19]. Moreover, among the antioxidant enzymes, the manganese-dependent superoxide dismutase (MnSOD) seems to be a major modulator in sensitising FRDA cells to death when exposed to oxidative stress [12,20,21].

The clinical picture of FRDA shows a life-threatening disease characterized by a multisystem, slowly progressive degeneration [22]. Most symptoms are the consequence of diffuse pathological processes affecting central and peripheral neurons in dorsal root ganglia, cerebral cortex and cerebellum, cardiomyocytes, and endocrine β-cells. FRDA patients thus develop a progressive gait and limb ataxia, subtle cognitive deficits, hypertrophic cardiomyopathy and high risk of diabetes mellitus. The search for effective therapeutic agents is ongoing, with a number of approaches at different stages of experimental and clinical evaluation. A group of current strategies are aimed at improving mitochondrial pathogenesis and/or reducing oxidative stress [16,21,23,24]. Treatments to increase frataxin expression include several approaches based on epigenetic modulators [25,26], drug repositioning [27,28,29,30,31], protein restoration [32,33], nucleic acid therapeutics [34,35] and gene replacement [36,37].

Among potential therapies, the cytokine interferon gamma (IFN-γ) has been shown to increase frataxin expression in FRDA cells. An animal model of FRDA demonstrated improvements in motor coordination, locomotion and dorsal root ganglia degeneration [38]. Subsequently, clinical studies reported a good safety profile and possible disease-modifying effects of IFN-γ in FRDA patients [39,40,41,42,43,44,45]. Today, a detailed mechanistic understanding of the pathway(s) targeted by this treatment in FRDA cells remains to be elucidated. IFN-γ is a pleiotropic cytokine that affects several biological processes, with the ability to regulate the expression of hundreds of genes [46]. Diverse in vitro and in vivo evidence intriguingly indicate that the treatment with IFN-γ confers cytoprotection to multiple cell types without a specific deficit of frataxin, such as normal primary neurons [47], microglial cells [48] and cardiomyocytes [49]. Therefore, the presence of pleiotropic pathways contributing to the beneficial effects of IFN-γ in FRDA, in addition to those leading to frataxin induction, cannot be ruled out. To investigate relevant molecular pathways triggered by IFN-γ, we focused our attention on cytoprotective players ensuring antioxidant responses that are known to be dysfunctional or unresponsive in FRDA cells.

## 2. Results

### 2.1. IFN-γ Exposure Upregulates Nrf2 and MnSOD Protein Levels in Human Cells with Normal and Defective Frataxin Expression

**Figure 1:**
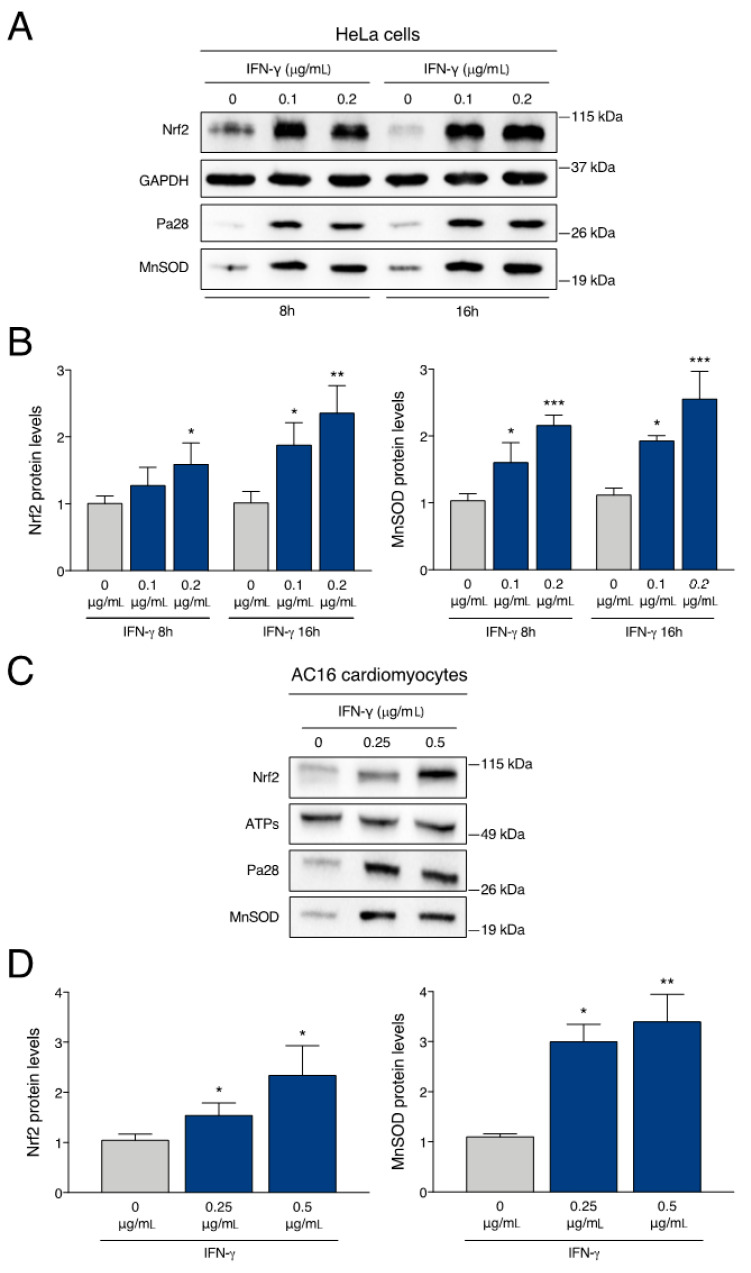
IFN-γ upregulates Nrf2 and MnSOD proteins in human cell lines. (**A**) Representative western blot analysis of HeLa cells treated with the indicated concentrations of IFN-γ (0.1 and 0.2 μg/mL) for 8 and 16 h. (**B**) Densitometric analysis of single protein levels in HeLa cells relative to untreated cells (0 μg/mL) and normalized with GAPDH levels. (**C**) Representative western blot analysis of human AC16 cardiomyocytes treated with the indicated concentrations of IFN-γ (0.25 and 0.5 μg/mL) for 16 h. (**D**) Densitometric analysis of single protein levels in AC16 cardiomyocytes relative to untreated cells (0 μg/mL) and normalized with ATP synthase levels. Densitometric data in (**B**,**D**) indicate the mean ± SD from three independent experiments. *p* values were calculated by one-way ANOVA with post-hoc comparison to untreated sample (*, *p* < 0.05; **, *p* < 0.01; ***, *p* < 0.001).

To identify molecular targets that are modified during the early phases of IFN-γ signalling, we focused our attention on cytoprotective proteins with potential relevance to FRDA pathophysiology. Because recent evidence suggest that IFN-γ can induce protective responses in normal cells [47,48,49], we initially performed our molecular analysis in a cellular model with natural frataxin expression. For this purpose, we used HeLa cells, a typical cellular system for the study of IFN-γ signalling [50]. HeLa cells were exposed to different doses of IFN-γ and analysed after 8 and 16 h. As shown in Figure 1, HeLa extracts were evaluated by western blotting for the expression of Nrf2, a transcription factor downregulated in cellular and animal models of FRDA [51], and of MnSOD, a mitochondrial antioxidant enzyme disabled in FRDA cells [12,20]. The levels of PA28a protein, a component of immunoproteasome typically induced by IFN-γ [52], were used as a positive control. In HeLa cells, expression levels of Nrf2 and MnSOD are optimally induced with 0.2 μg/mL IFN-γ, appearing upregulated by about 2.5-folds after 16 h exposure. As expected, PA28a is also strongly upregulated.

We then replicated the analysis in a different, disease-relevant cell line. The most affected cell types in FRDA are neurons and cardiomyocytes. Indeed, heart failure is the main cause of premature death in FRDA patients. To evaluate the response of cardiac cells to IFN-γ, we exposed the AC16 human cardiomyocytes to different doses of IFN-γ and analysed the cellular extracts by western blotting after 16 h. Similarly to what observed with HeLa cells, the levels of Nrf2 and MnSOD are strongly upregulated in AC16 cells, after the treatment with IFN-γ (Figure 1C,D).

**Figure 2:**
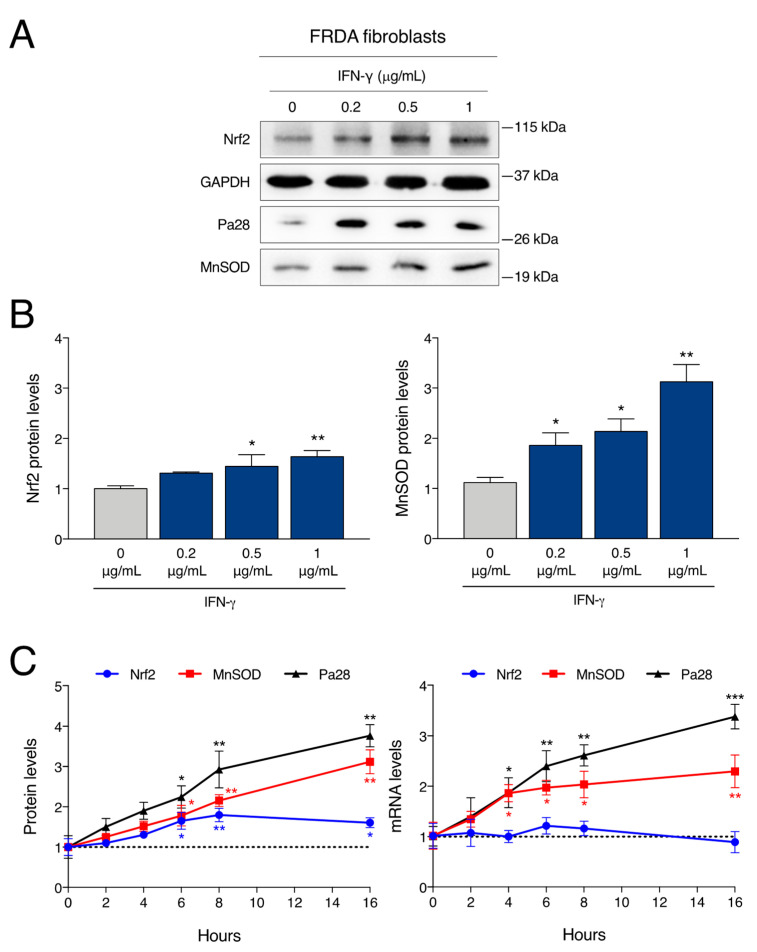
IFN-γ enhances Nrf2 and MnSOD expression in FRDA cells. Primary FRDA fibroblasts were treated with the indicated concentrations of IFN-γ (0.2, 0.5 and 1 μg/mL) for 16 h. (**A**) Representative western blot analysis. (**B**) Densitometric analysis of single protein levels relative to untreated cells (0 μg/mL) and normalized with GAPDH levels. Densitometric data indicate the mean ± SD from four independent experiments. *p* values were calculated by one-way ANOVA with post-hoc comparison to untreated sample (*, *p* < 0.05; **, *p* < 0.01). (**C**) Time-course expression analysis of primary FRDA fibroblasts after treatment with 1 μg/mL of IFN-γ. Left panel: western blot densitometric analysis for the levels of Nrf2, MnSOD and Pa28 proteins. Right panel: qRT-PCR analysis for the levels of Nrf2, MnSOD and Pa28 mRNAs. Data indicate the mean values ± SD from three independent experiments; each value is relative to untreated cells at the same time-point. *p* values were calculated by one-way ANOVA with post-hoc comparison to untreated sample (*, *p* < 0.05; **, *p* < 0.01; ***, *p* < 0.001).

We thus investigated whether IFN-γ is able to elicit the same effects also in FRDA patient-derived cells. Human primary FRDA fibroblasts were treated with IFN-γ for 16 h in dose-response experiments and evaluated for expression of the target proteins. Again, exposure to IFN-γ enhances the expression levels of Nrf2 and MnSOD in a dose-dependent fashion, peaking at 1 μg/mL (Figure 2A,B).

### 2.2. IFN-γ Activates Two Distinct Pathways to Induce Nrf2 and MnSOD Expression in FRDA Cells

To gain insight into the mechanism activating the expression of Nrf2 and MnSOD, we sought to discriminate between possible transcriptional and post-transcriptional regulation events in response to IFN-γ. We thus analysed the kinetics of protein and mRNA induction by time-course experiments in FRDA fibroblasts. Cells were treated with IFN-γ for between 2 and 16 h to obtain the protein and RNA extracts at selected time points. As shown in Figure 2C, IFN-γ induces a sustained and time-dependent upregulation of MnSOD protein levels, already evident after 4 h. In contrast, the Nrf2 protein levels displayed a slower rise, peaking after about 8 h. To examine the corresponding transcript levels, *NFE2L2* (Nrf2) and *SOD2* (MnSOD) mRNAs were assessed by quantitative RT-PCR analysis (Figure 2C). The MnSOD mRNA is strongly upregulated by the treatment with IFN-γ, in a time-dependent fashion. Positive control *PSME1* (PA28a) mRNA shows a similar pattern, i.e., a quick and progressive increase. Conversely, the levels of Nrf2 mRNA show only minor fluctuations, with no increase over time. Notably, the upregulation of MnSOD mRNA can be appreciated before the significant accumulation of Nrf2 protein, suggesting that Nrf2 is not involved in the transcriptional activation of MnSOD. Altogether, these results suggest that two different pathways are activated by IFN-γ: an early transcriptional regulation of MnSOD and a late post-transcriptional regulation of Nrf2.

### 2.3. IFN-γ Treatment Enhances the p21-Nrf2 Pathway in FRDA Cells

**Figure 3:**
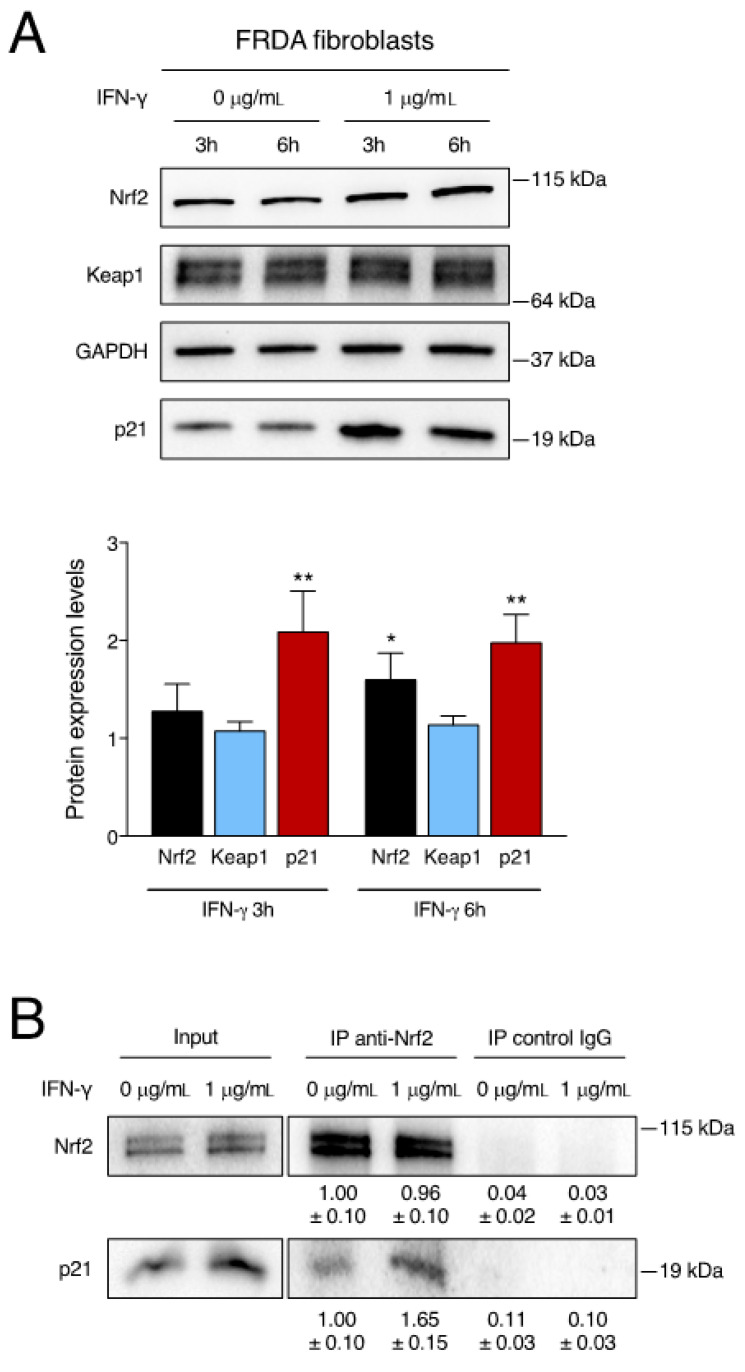
IFN-γ induces the p21/Nrf2 pathway in FRDA cells. (**A**) Primary FRDA fibroblasts were left untreated or treated with 1 μg/mL of IFN-γ. Upper panel: Representative western blot analysis of Nrf2, Keap1 and p21 protein expression. Lower panel: Densitometric analysis of Nrf2, Keap1 and p21 protein levels relative to untreated cells (0 μg/mL) and normalized with GAPDH levels. Densitometric data indicate the mean ± SD from three independent experiments. *p* values were calculated by one-way ANOVA with post-hoc comparison to untreated sample (*, *p* < 0.05; **, *p* < 0.01). (**B**) Primary FRDA fibroblasts were left untreated (0 μg/mL) or treated with 1 μg/mL of IFN-γ for 3 h. Cell extracts were immunoprecipitated with anti-Nrf2 antibody (IP anti-Nrf2) or with an irrelevant isotype-matching antibody (IP control IgG) and immunoblotted with anti-Nrf2 antibody or anti-p21 antibody. The densitometric quantitation of immunoprecipitated proteins is indicated as the mean ± SD from three independent experiments.

To elucidate the induction of Nrf2, we investigated the post-transcriptional mechanism triggered by IFN-γ. Under physiological conditions, the main control of Nrf2 comes from the interaction with Keap1, which drives its basal ubiquitin-proteasome-mediated degradation. Downregulation of Keap1, in turn, allows Nrf2 stabilization and accumulation [53]. Thus, FRDA fibroblasts were treated with IFN-γ and evaluated by western blotting for the levels of Nrf2 and Keap1 in the first hours of stimulation. As shown in Figure 3A, an effective dose of IFN-γ induces significant Nrf2 accumulation at 6 h. However, the treatment with IFN-γ does not cause any reduction of Keap1 protein levels in FRDA fibroblasts and thus does not justify the stabilization and accumulation of Nrf2. Because specific proteins have the ability to destabilize the Keap1-Nrf2 complex by direct interaction, we examined the levels of p21 protein, known to play a key role in stabilizing Nrf2 expression [54]. The results indicate that treatment of FRDA fibroblasts with IFN-γ triggers a strong p21 upregulation that is already significant after 3 h, thus preceding optimal Nrf2 accumulation (Figure 3A). To further examine the p21-Nrf2 pathway, we analysed the physical interaction between p21 and Nrf2 in FRDA fibroblasts in the presence or absence of IFN-γ. Immunoprecipitation experiments confirmed that p21 interacted with Nrf2 in untreated cells. More importantly, the binding of p21 to Nrf2 was greatly enhanced when FRDA fibroblasts were stimulated by IFN-γ (Figure 3B).

### 2.4. IFN-γ Potentiates Oxidative Stress Response in FRDA Cells

**Figure 4:**
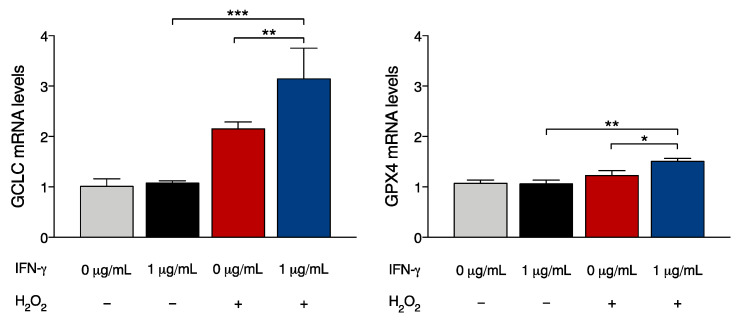
The presence of IFN-γ during oxidative stress potentiates Nrf2 downstream transcripts in FRDA cells. Primary FRDA fibroblasts were left untreated (0 μg/mL) or treated with 1 μg/mL IFN-γ, 600 μM hydrogen peroxide (H_2_O_2_) or cotreated with IFN-γ and H_2_O_2_ for 6 h. The expression levels of *GCLC* and *GPX4* mRNAs were analysed by qRT-PCR. Data indicate the mean ± SD from three independent experiments; *p* values were calculated by one-way ANOVA with post-hoc comparison (*, *p* < 0.05; **, *p* < 0.01; ***, *p* < 0.001).

Because the induction of Nrf2 and MnSOD potentially grants cytoprotective responses, we evaluated if the treatment with IFN-γ effectively modifies pathophysiological defects of FRDA patient cells. To deepen the significance of upregulated Nrf2 levels following IFN-γ treatment, we evaluated the expression of Nrf2 target transcripts under basal and oxidative conditions (Figure 4). Quantitative RT-PCR analysis in FRDA fibroblasts exposed to IFN-γ showed no effect on the induction of Glutamate-Cysteine Ligase Catalytic Subunit (*GCLC*) or Glutathione Peroxidase 4 (*GPX4*) transcripts. These results suggest that, in basal conditions, IFN-γ allows Nrf2 stabilization and accumulation but does not trigger its transcriptional activity in FRDA fibroblasts. We then evaluated the response under oxidative conditions. *GCLC* and *GPX4* mRNAs were analysed after exposure to hydrogen peroxide and showed significant upregulation, as expected. Interestingly, co-treatment of FRDA fibroblasts with IFN-γ and hydrogen peroxide revealed enhancing effects on the expression of *GCLC* and *GPX4* transcripts, with a significantly higher induction over the single exposure to hydrogen peroxide (Figure 4).

**Figure 5:**
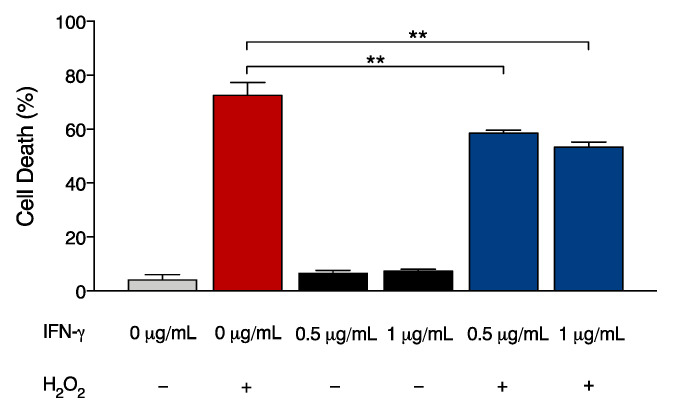
IFN-γ reduces hydrogen peroxide-induced cell death in FRDA cells. FRDA fibroblasts were left untreated (0 μg/mL) or treated with the indicated doses of IFN-γ (0.5 and 1 μg/mL), 800 μM hydrogen peroxide (H_2_O_2_) or cotreated with IFN-γ and H_2_O_2_. Cell death was determined after 5 h by trypan blue assay. Data indicate the mean ± SD from three independent experiments. *p* values were calculated by one-way ANOVA with post-hoc comparison to H_2_O_2_ sample (**, *p* < 0.01).

We therefore evaluated whether the treatment with IFN-γ was able to modify the sensitivity of FRDA fibroblasts toward oxidative stress. To this end, FRDA cells were exposed to death-inducing doses of hydrogen peroxide either in the presence or absence of IFN-γ. The results in Figure 5 demonstrate that the stimulation by IFN-γ significantly reduces hydrogen peroxide-induced cell death in FRDA fibroblasts. Collectively, our data indicate that early pathways arising from IFN-γ stimulation contribute to potentiate cytoprotective and antioxidant activities in FRDA cells.

## 3. Discussion

All the metabolic defects affecting FRDA cells are thought to be a domino effect initiated by insufficient activity of frataxin. Indeed, defective ISC biosynthesis, mitochondrial iron accumulation, reduced efficiency of oxidative phosphorylation and increased ROS production are dysfunctions strictly linked to each other [55]. In particular, oxidative damage in FRDA is related to impaired expression and/or activity of the transcription factor Nrf2, the key mediator of antioxidant defences [56]. Albeit a disorganization of the cytoskeleton in FRDA cells [17] and an upregulation of the Nrf2 inhibitor Keap-1 in a FRDA mouse model [57] have been specifically involved in this disease, the molecular mechanism linking frataxin depletion to Nrf2 disablement still remains to be understood. Likewise, shortfall of superoxide dismutases (SODs) is associated with the increased sensitivity to oxidative stress in FRDA. These antioxidant enzymes are typically induced in the presence of pro-oxidants in order to eliminate superoxide free radicals within the mitochondrial and cytosolic compartments. However, FRDA cells lose the physiological ability of healthy cells to upregulate SODs when exposed to an oxidative insult [12,20] or to maintain protective basal expression levels [13,58].

In this study, we explored the activation of early cytoprotective pathways arising from IFN-γ exposure and potentially relevant in FRDA pathophysiology. IFN-γ represents a potential therapy under clinical evaluation in FRDA patients [39,40,42,43]. In this regard, we previously described that IFN-γ induces a transcriptional activation of *FXN* gene leading to frataxin protein upregulation after 24 h exposure [38]. However, earlier events triggered by IFN-γ could significantly contribute to its pharmacological action. Indeed, IFN-γ is a cytokine potentially able to activate multiple signalling pathways and gene expression profiles. Importantly, IFN-γ can widely affect cell physiology and survival by epigenetic regulations, modulation of transcription factors and activation of protein kinase networks [59]. Our results demonstrate for the first time in FRDA cells that IFN-γ is consistently able to induce, in the first hours of exposure, the expression of Nrf2 and mitochondrial MnSOD. A single study reported that IFN-γ is able to stimulate the Nrf2 pathway during classical macrophage activation [60]. However, both the molecular mechanism and the effects of this IFN-γ-Nrf2 axis are entirely unexplored in FRDA cells. Under physiological conditions, the main control of Nrf2 comes from degradation by the ubiquitin-proteasome system, distribution in different cell compartments and post-translational modifications [53]. Moreover, activation of Nrf2 by transcriptional regulation was also described. The transcription factors NF-κB [61], AhR [62], Jun and Myc [63] increase Nrf2 mRNA and protein levels. To understand the mechanism involved, we examined the Nrf2 and MnSOD induction kinetics following IFN-γ stimulation in FRDA cells. As IFN-γ signalling typically results in the initiation of interferon-stimulated gene transcription, we first focused on the evaluation of transcriptional expression. In fact, under certain circumstances, a direct upregulation of MnSOD following the activation of Nrf2 was described [64]. Our results indicate a very rapid transcriptional regulation of the MnSOD transcript within 2–4 h, and no transcriptional effect on Nrf2 mRNA. Furthermore, analysis of the corresponding kinetics of protein expression showed a delayed accumulation of Nrf2 protein. Therefore, our data indicate that in FRDA cells IFN-γ activates two distinct pathways to enhance Nrf2 and MnSOD levels and do not suggest a direct role of Nrf2 in the transcriptional regulation of MnSOD. By investigating the post-transcriptional regulation of Nrf2, we revealed for the first time that IFN-γ can stimulate a non-canonical Nrf2 activation pathway [65] through the upregulation of p21 and enhanced formation of Nrf2/p21 complex [54]. Moreover, while treatment of FRDA fibroblasts with IFN-γ potentiated Nrf2 expression, this was insufficient to induce antioxidant transcripts under basal conditions. Notably, the exposure to hydrogen peroxide showed the ability to elevate expression of *GCLC* and *GPX4* downstream transcripts in FRDA fibroblasts; however, the degree of induction was significantly higher after co-treatment with hydrogen peroxide and IFN-γ. Not surprisingly, this agrees with the fact that p21 directly interacts with the DLG motif in Nrf2: this binding inhibits Nrf2 degradation but without disrupting the cytosolic complex with Keap1 [54,66]. Thus, we speculate that oxidative stress is required to unlock the transcription of antioxidant genes by the Nrf2 pool accumulated in FRDA fibroblasts through IFN-γ stimulation. The ability of IFN-γ to recruit antioxidant players in FRDA fibroblasts prompted us to assess if the treatment effectively grants cytoprotective responses. A large body of data demonstrated the enhanced sensitivity of cellular and animal FRDA models to a variety of pro-oxidants [67]. To this purpose, we evaluated the rescue of oxidative-stress response in FRDA fibroblasts. Notably, our results demonstrate that IFN-γ significantly reduces the sensitivity to hydrogen peroxide-induced cell death. Altogether, we can suggest that IFN-γ is effective in counteracting a major dysfunctional outcome of FRDA through an early recruitment of cytoprotective pathways.

The superoxide-scavenging activity of MnSOD is of great relevance to deleterious FRDA alteration in iron homeostasis [68]. Indeed, mitochondrial reactive iron can potentiate the oxidative stress in FRDA by producing superoxide and hydrogen peroxide via the Fenton chemistry [69]. Without invoking the FRDA context, several studies concluded that MnSOD overexpression prevents cell death and reactive oxidant generation in neuronal and cardiac cells [70,71,72]. Interestingly, a previous paper demonstrated the ability of IFN-γ to protect microglia from various reactive species through a selective upregulation of MnSOD [48].

The potential role of Nrf2 is clearly more wide-ranging. This master transcriptional factor can target the expression of about 250 genes that contain the antioxidant response element (ARE) regulatory regions [19]. Consequently, activation of Nrf2 leads to higher levels of cytoprotective proteins, including detoxifying and antioxidants enzymes, anti-apoptotic proteins, mitochondrial proteins and other transcription factors. It is now widely recognized that Nrf2 is essential for protection against cardiovascular and neurological diseases that have oxidative stress and altered mitochondrial homeostasis as underlying pathological feature. A number of natural and chemical Nrf2 activators are now at various stages of clinical development [73]. Recent studies demonstrated the efficacy of Nrf2-activating drugs in cellular and animal model of FRDA [16,27,74]. More importantly, on February 2023 the U.S. Food and Drug Administration has approved the Nrf2 activator Omaveloxolone [75] as the first therapy indicated to treat FRDA patients aged 16 years and over [76]. Besides the multiple antioxidant effects, some Nrf2 inducers also increase the transcription of *FXN* gene in FRDA cells. Indeed, the presence of ARE target regions was demonstrated in the *FXN* locus [27]. However, most but not all Nrf2 inducers have the ability to upregulate frataxin expression [77]. Nevertheless, the beneficial effects of Nrf2 activation appear equally evident in FRDA cells, as typified by omaveloxolone [16]. Although appealing, the involvement of Nrf2 as direct effector in the mechanism of frataxin upregulation by IFN-γ seems unlikely. The kinetic of frataxin mRNA in FRDA fibroblasts, exposed to the same dose of IFN-γ used in the present study, is characterized by a quick rise within 2 h from treatment and succeeding return to basal levels at 4 h [38]. As discussed about the early transcriptional effect of IFN-γ on MnSOD mRNA, no significant increase of Nrf2 was detected in FRDA fibroblasts before 4 h exposure. Thus, such a sequence of molecular events can hardly explain the induction of frataxin gene transcription.

Collectively, the results of the present study suggest the presence of pleiotropic pathways contributing to the therapeutic relevance of IFN-γ in FRDA, besides those leading to frataxin induction. Future studies on the synergic action between early and late molecular players could help in the design of better therapeutic schedules and shed light on novel therapeutic targets for FRDA.

## 4. Materials and Methods

### 4.1. Cell Culture and Treatment

HeLa cells were obtained from ECACC, Salisbury, UK (Cat. No. 93021013) and maintained in culture with Dulbecco’s Modified Eagle’s Medium (DMEM Euroclone ECB7501, Pero, Italy) supplemented with 10% fetal bovine serum heat inactivated (FBS Hyclone CHA1115L, Logan, UT, USA), 100 U/mL penicillin + 0.1 mg/mL streptomycin (Euroclone ECB3001), and 2 mM L-glutamine (Euroclone ECB3000). AC16 human cardiomyocyte cell line from Merck Millipore, Burlington, MA, USA (Cat. No. SCC109) were cultured in DMEM-F12 medium (Gibco 11320033, Thermo Fisher Scientific, Waltham, MA, USA), supplemented with 12.5% FBS heat inactivated, penicillin/streptomycin, and 2 mM L-glutamine. Human primary fibroblasts were obtained from NIGMS Human Genetic Repository, Coriell Institute for Medical Research, Camden, NJ, USA. GM03816 fibroblasts derive from a clinically affected FRDA patient homozygous for the GAA expansion in the *FXN* gene with alleles of approximately 330 and 380 repeats. FRDA fibroblasts were maintained in culture in DMEM supplemented with 15% FBS heat inactivated, penicillin/streptomycin and 2 mM L-glutamine.

Cells were treated with recombinant human IFN-γ (PeproTech 300-02, Cranbury, NJ, USA) reconstituted in sterile bidistilled water and further diluted in Dulbecco’s Phosphate Buffered Saline (DPBS Euroclone ECB4004), containing 10% FBS heat inactivated. Hydrogen peroxide solution 30% (*w*/*w*) (H1009 Sigma-Aldrich, Burlington, MA, USA) was freshly diluted in DPBS before each treatment. The untreated control cultures were incubated with equal volume of the solvent used to deliver test compounds.

### 4.2. Western Blot

Whole extracts from cells untreated and treated with recombinant human IFN-γ (PeproTech 300-02) or hydrogen peroxide (Sigma-Aldrich H1009) were prepared in ice-cold CelLytic M buffer (Sigma-Aldrich) supplemented with Complete Protease Inhibitor Cocktail (Roche Diagnostics, Monza, Italy). Amounts of 20–40 μg of protein extracts were analysed by western blotting with the following antibodies: mAb anti-Nrf2 (Abcam ab62352, Cambridge, UK), mAb anti-Pa28 (Enzo Life Sciences BML-PW8185, Farmingdale, NY, USA), mAb anti-mnSOD (Enzo Life Sciences ADI-SOD-110-F), mAb anti-p21 (Cell Signaling Technology 12D1, Danvers, MA, USA), mAb anti-Keap1 (Abcam 1B4 ab119403), mAb anti-GAPDH (Santa-Cruz sc-365062, Dallas, TX, USA), mAb anti-ATP synthase β (BD Biosciences 612519, Franklin Lakes, NJ, USA), and secondary antibody horseradish peroxidase (HRP)-conjugated goat anti-mouse or mouse anti-rabbit (Thermo Fisher Scientific). Digital images acquisition and densitometric analysis were performed using a ChemiDoc XRS system (Bio-Rad, Hercules, CA, USA) equipped with the ImageLab 5.2.1 software (Bio-Rad). The uncropped western blot images are provided in the Supplementary Figures S1–S4.

### 4.3. Immunoprecipitation

Whole cell extracts were prepared and processed for immunoprecipitation using a Pierce Classic IP Kit (Thermo Fisher Scientific 26146). For preparation and capture of immune complexes, 1 mg of whole cell lysates was incubated following the manufacturing instructions with 2 μg of mAb anti-Nrf2 (Abcam ab62352) to immunoprecipitate Nrf2 protein, or with 2 μg of mAb anti-GPX4 (Abcam ab125066) as an isotype-matched negative control. Immunocomplexes were resuspended in Lane Marker Sample Buffer 2X (Thermo Fisher Scientific 39000). Samples were then boiled for 5 min, resolved by SDS-PAGE, and analysed by western blotting as above described.

### 4.4. RNA Isolation, Reverse Transcription (RT) and Quantitative RT-PCR (qRT-PCR)

Total RNA extraction and cDNA synthesis were performed as previously described [35]. The mRNA expression levels were assessed by real-time qPCR employing the following cycling conditions: 1 cycle at 50 °C for 2 min and 95 °C for 10 min; 40 cycles at 95 °C for 15 s and 60 °C for 1 min. The qRT-PCR reactions were carried out with the following predesigned primers and TaqMan probes (TaqMan Gene Expression Assays) from Thermo Fisher Scientific: NFELE2 (Nrf2) Hs00975960_m1; SOD2 (MnSOD) Hs00167309_m1; PSME1 (Pa28) Hs00389209_m1; GCLC Hs00155249_m1; GPX4 Hs00157812_m1; hGAPDH, Hs99999905_m1; GUSB Hs00939627_m1; and ACTB Hs99999903_m1. GAPDH, GUSB and ACT were used as reference genes for normalization. Levels of human PA28a mRNA were checked as positive control of IFN-γ stimulation. The amplified transcripts were quantified using the comparative Ct method and the differences in gene expression were presented as normalized fold expression with ΔΔCt method.

### 4.5. Measurement of Cell Viability by Trypan Blue Assay

For cell viability assay, human GM03816 FRDA fibroblasts were cultured in 12-well plates by seeding 0.6 × 10^5^ cells per well. Cells were left untreated or treated with IFN-γ. After incubation in presence of 800 μM hydrogen peroxide for 5 h, fibroblasts from each incubation point were collected as follows: floating cells in culture supernatant were recovered into a centrifuge tube and pooled to the same tube with adherent cells detached by incubation at 37 °C in Trypsin-EDTA 1× (Euroclone ECB3052). After centrifugation at 800× *g* for 10 min (4 °C), cell pellet was resuspended in DPBS and cell death was analysed by Trypan Blue assay using a Countess Automated Cell Counter (Thermo Fisher Scientific).

### 4.6. Statistical Analysis

The significance of differences between groups was evaluated by one-way ANOVA with the following post hoc comparisons: Dunnett’s test to compare every mean to a control mean and Tukey’s test to compare every mean with every other mean. In all experiments, *p <* 0.05 was considered significant. Quantitative data are presented as the mean ± SD of at least three independent experiments.

## Data Availability

All data generated or analysed during this study are included in this published article.

## Supplementary Materials

The following supporting information can be downloaded at: https://www.mdpi.com/article/10.3390/ijms241612687/s1.
