# Supplementary material for: Interferon Gamma Enhances Cytoprotective Pathways via Nrf2 and MnSOD Induction in Friedreich’s Ataxia Cells
Source: Int J Mol Sci. 2023 Aug 11;24(16):12687. doi: 10.3390/ijms241612687 (PMC10454386; doi:10.3390/ijms241612687)
Supplement: Supplementary file 1 [file ijms-24-12687-s001.zip › ijms-2468635-supplementary.pdf]

Interferon gamma enhances cytoprotective pathways via Nrf2 and MnSOD induction in Friedreich's ataxia cells

Riccardo Luffarelli[1,†], Luca Panarello[1,†], Andrea Quatrana[1], Francesca Tiano[1], Silvia Fortuni[1], Alessandra Rufini[1,2], Florence Malisan[1], Roberto Testi[1] and Ivano Condò[1*]

[1] Department of Biomedicine and Prevention, University of Rome Tor Vergata, 00133 Rome, Italy.

[2] Departmental Faculty of Medicine and Surgery, Saint Camillus International University of Health and Medical Sciences, 00131 Rome, Italy.

* Correspondence: ivano.condo@uniroma2.it

† These authors contributed equally to this work.

SUPPLEMENTARY FIGURES

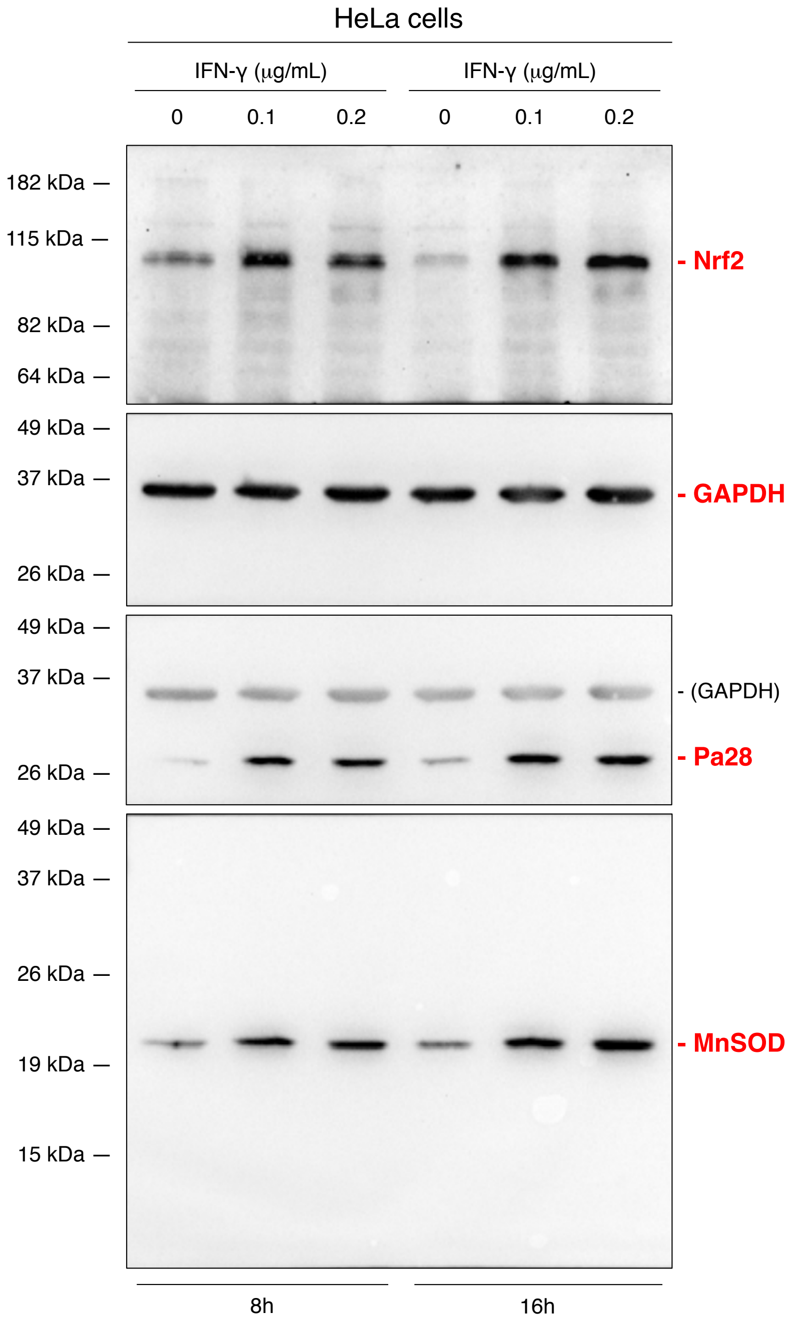

Figure S1. Uncropped western blot images of Fig. 1A showing Nrf2, GAPDH, Pa28 and MnSOD expression in HeLa cells.

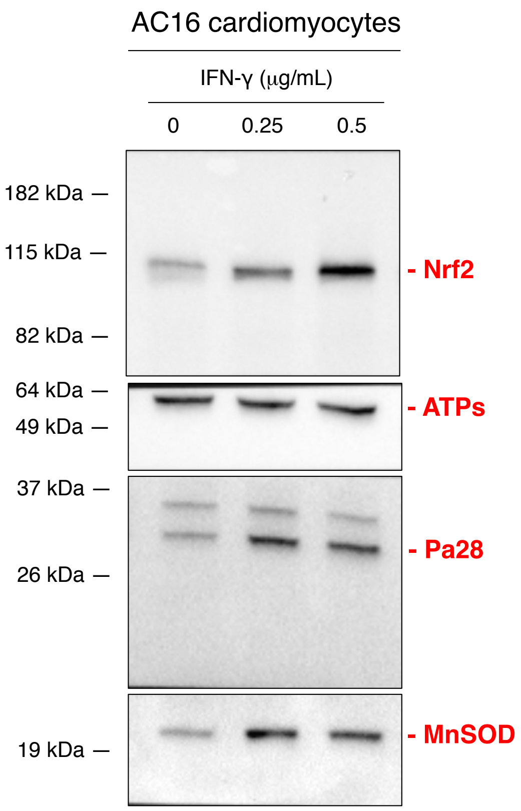

Figure S2. Uncropped western blot images of Fig. 1C showing Nrf2, ATP synthase, Pa28 and MnSOD expression in AC16 cardiomyocyte cells.

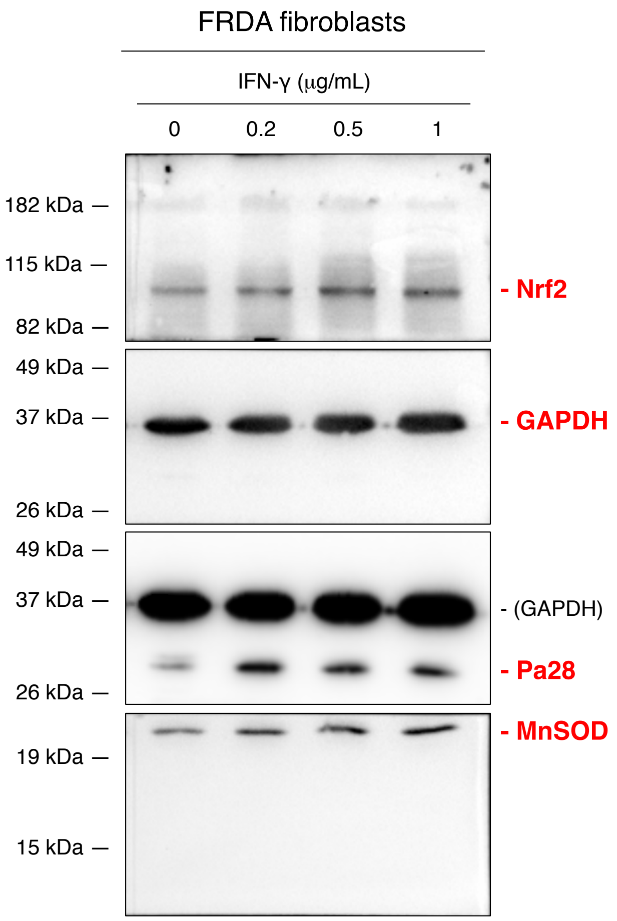

Figure S3. Uncropped western blot images of Fig. 2A showing Nrf2, GAPDH, Pa28 and MnSOD expression in FRDA fibroblasts.

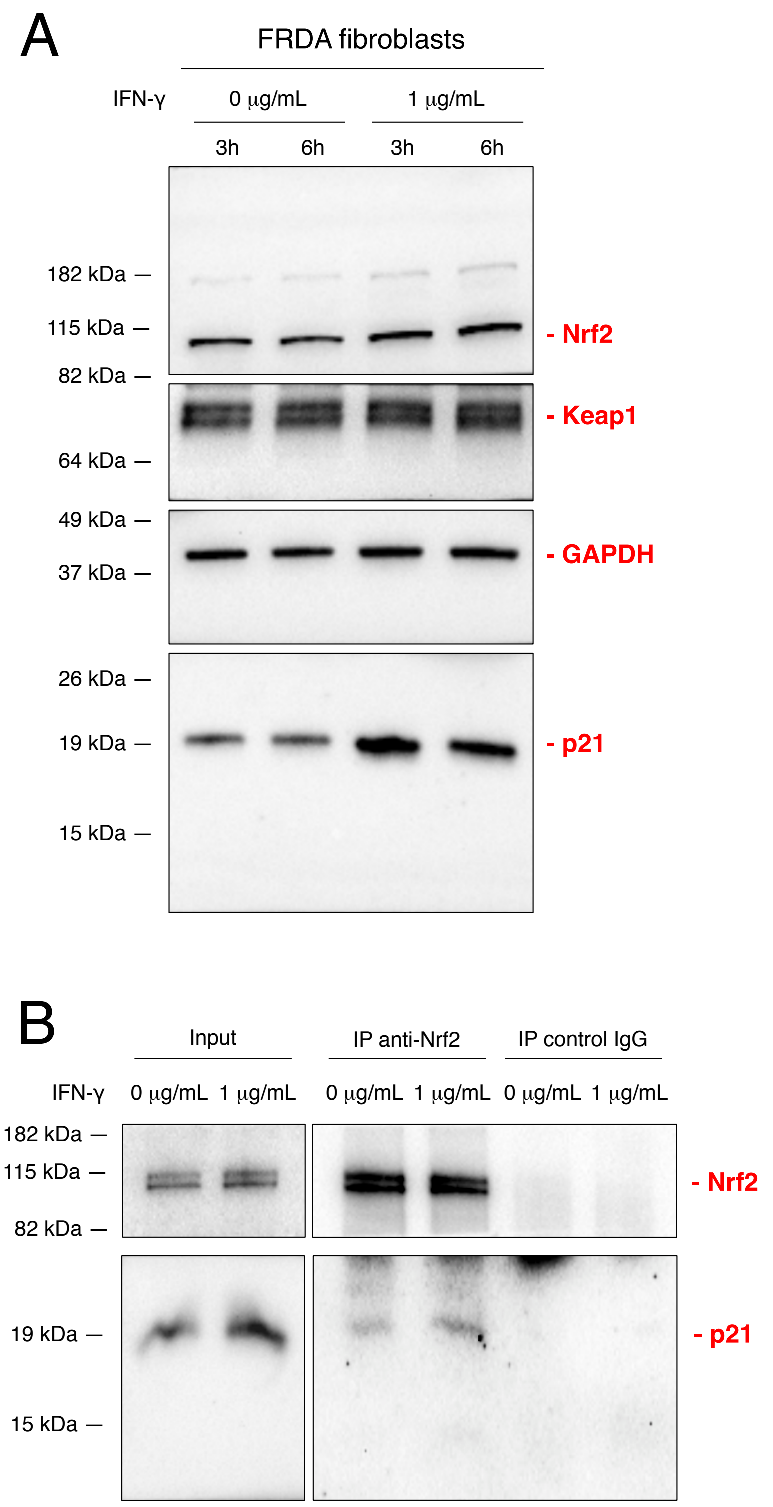

Figure S4. (**A**) Uncropped western blot images of Fig. 3A showing Nrf2, Keap1, GAPDH and p21 expression in FRDA fibroblasts. (**B**) Uncropped western blot images of Fig. 3B showing Nrf2 and p21 in whole cell extracts and immunoprecipitated samples from FRDA fibroblasts.
